# Supplementary figures and images for: Collagen cross-links scale with passive stiffness in dystrophic mouse muscles, but are not altered with administration of a lysyl oxidase inhibitor
Source: PLoS One. 2022 Oct 27;17(10):e0271776. doi: 10.1371/journal.pone.0271776 (PMC9612445; doi:10.1371/journal.pone.0271776)

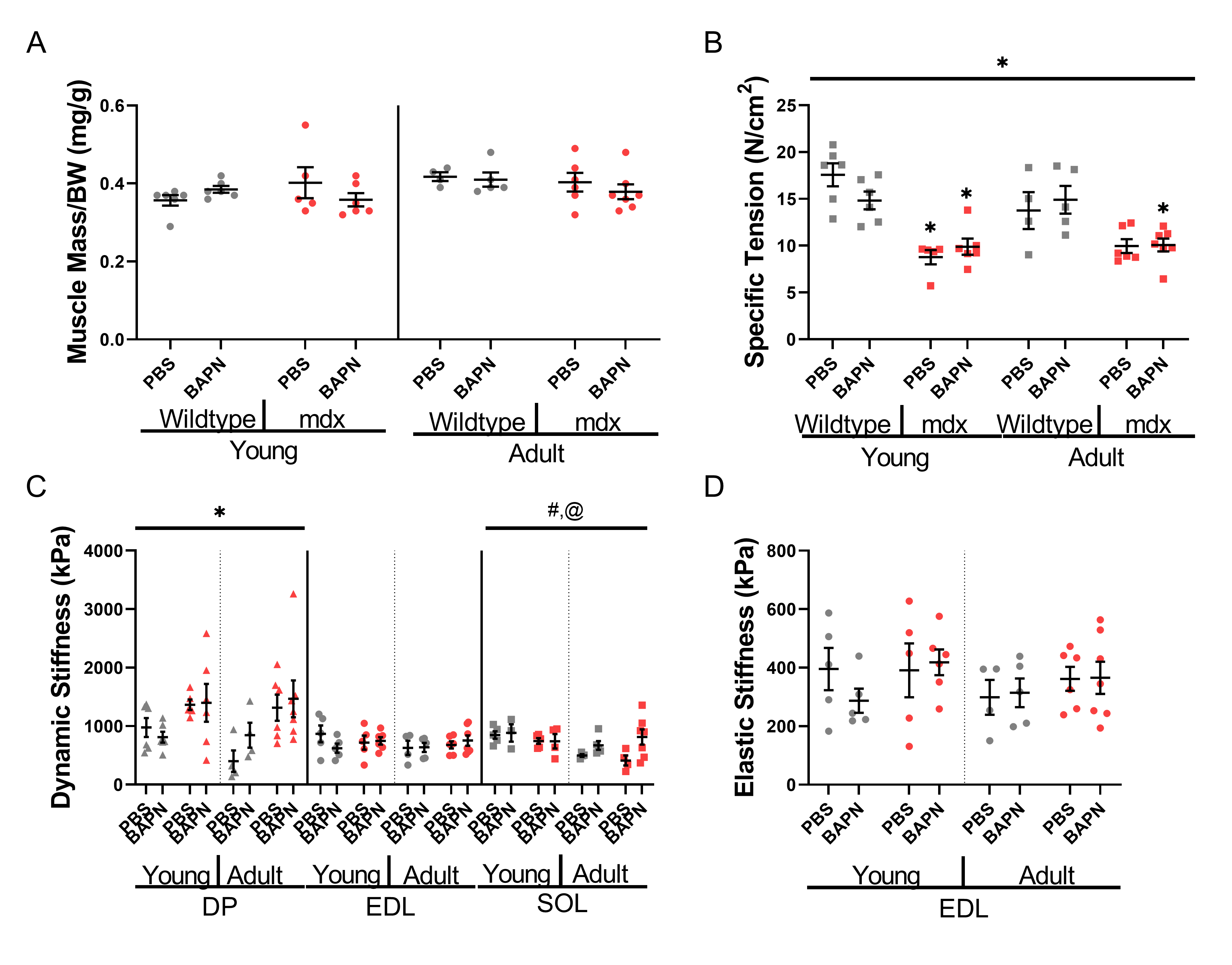

Supplement: S1 Fig — Mdx EDL showed a reduction in specific tension with no change in elastic stiffness. (A) There was no significant effect of genotype, age, or treatment on muscle weight in the EDL. While the standard mdx model often shows a pseudohypertrophy of limb muscle, the D2.mdx mouse muscle does not experience the increase in muscle mass [27]. (B) Specific tension during a maximal tetanus was reduced in the mdx EDL compared to wildtype. (C) Dynamic stiffness was increased in mdx in the diaphragms and was also increased in the young soleus compared to adult. There was no main effect of treatment, age, or genotype on the EDL dynamic stiffness. (D) There was no main effect of treatment, age, or genotype on the EDL elastic stiffness. * = genotype effect, # = age effects, @ = treatment effects, * = p<0.05 determined by two-way ANOVAs with post-hoc Sidak multiple comparisons tests. (TIF) [file pone.0271776.s001.tif]

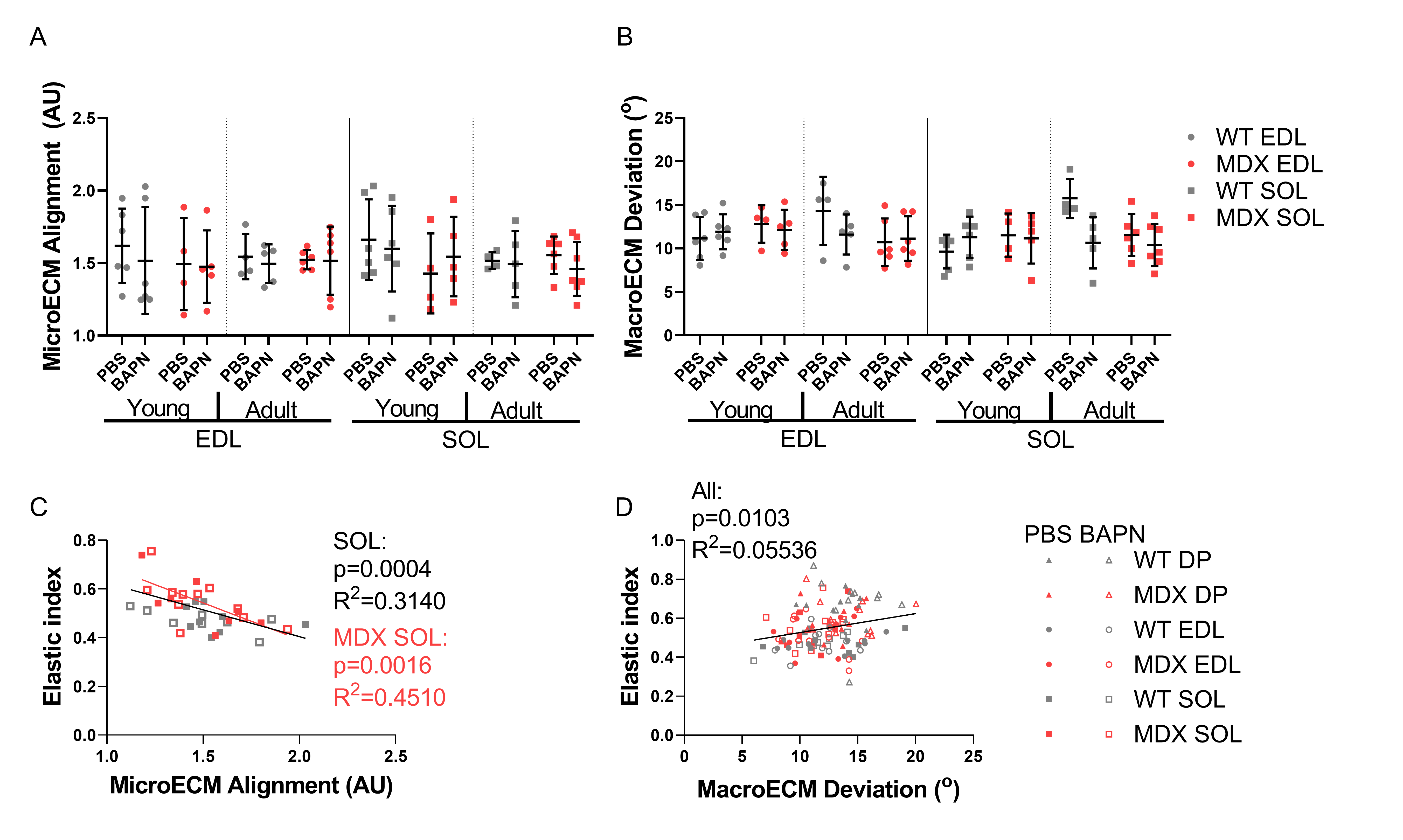

Supplement: S2 Fig — (A-B) There were no significant main effects determined by three-way ANOVAs across treatment, genotype, and age for EDL and soleus muscle MicroECM alignment and MacroECM deviation. (C) MicroECM alignment of overall and mdx soleus muscles scaled negatively with elastic index. (D) MacroECM deviation demonstrated a weak positive correlation with elastic index across all muscles. (TIF) [file pone.0271776.s002.tif]

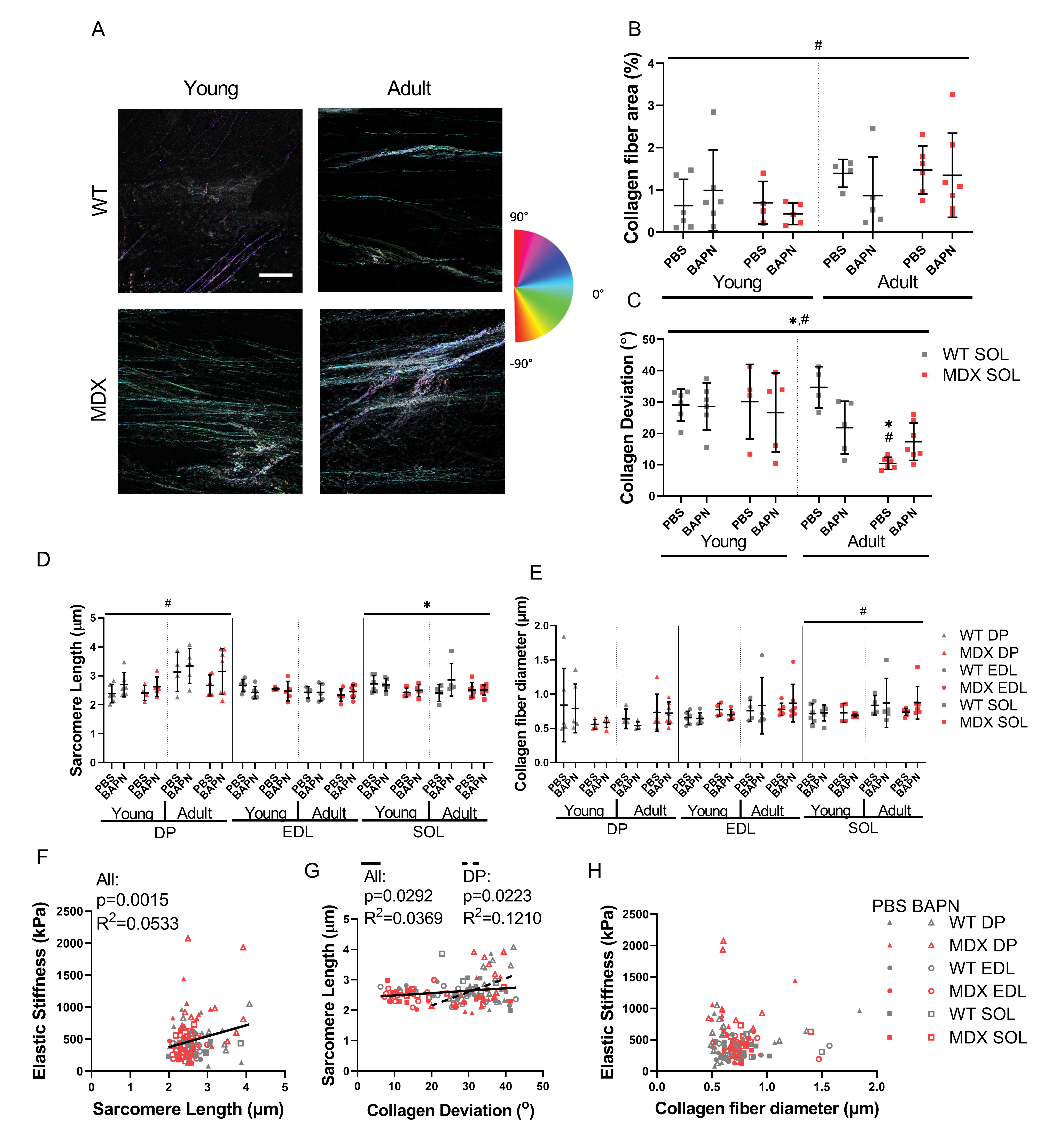

Supplement: S3 Fig — Mdx soleus has greater collagen alignment and sarcomere length relates to elastic stiffness. (A) Second Harmonic Generation (SHG) representative images of soleus muscle sections from young, adult, wildtype, and mdx mice. Colormap represents angles of each pixel window in the images as analyzed by OrientationJ. Scale bar is 100μm. (B) Adult solei had higher collagen fiber area than young solei. (C) Adult and mdx solei had lower collagen deviation (higher alignment) than young and wildtype solei. (D) Adult diaphragm muscles had longer sarcomere lengths than young diaphragms, and wildtype solei had longer sarcomere lengths than mdx. (E) Adult soleus muscles had larger mean collagen fiber diameters than young soleus muscles. (F) Collagen deviation had a positive correlation with sarcomere length across all muscles and in the diaphragm. (G) There was a significant positive correlation between sarcomere length and elastic stiffness across all groups. (H) There was no significant correlation between collagen fiber diameter and elastic stiffness. Significance bars represent significant main effects determined by three-way ANOVAs for treatment, genotype, and age. Significance symbol above groups indicates significance from corresponding genotype or age according to post-hoc Sidak multiple comparisons tests. Significance by genotype: *p<0.05 and age: #p<0.05. (TIF) [file pone.0271776.s003.tif]

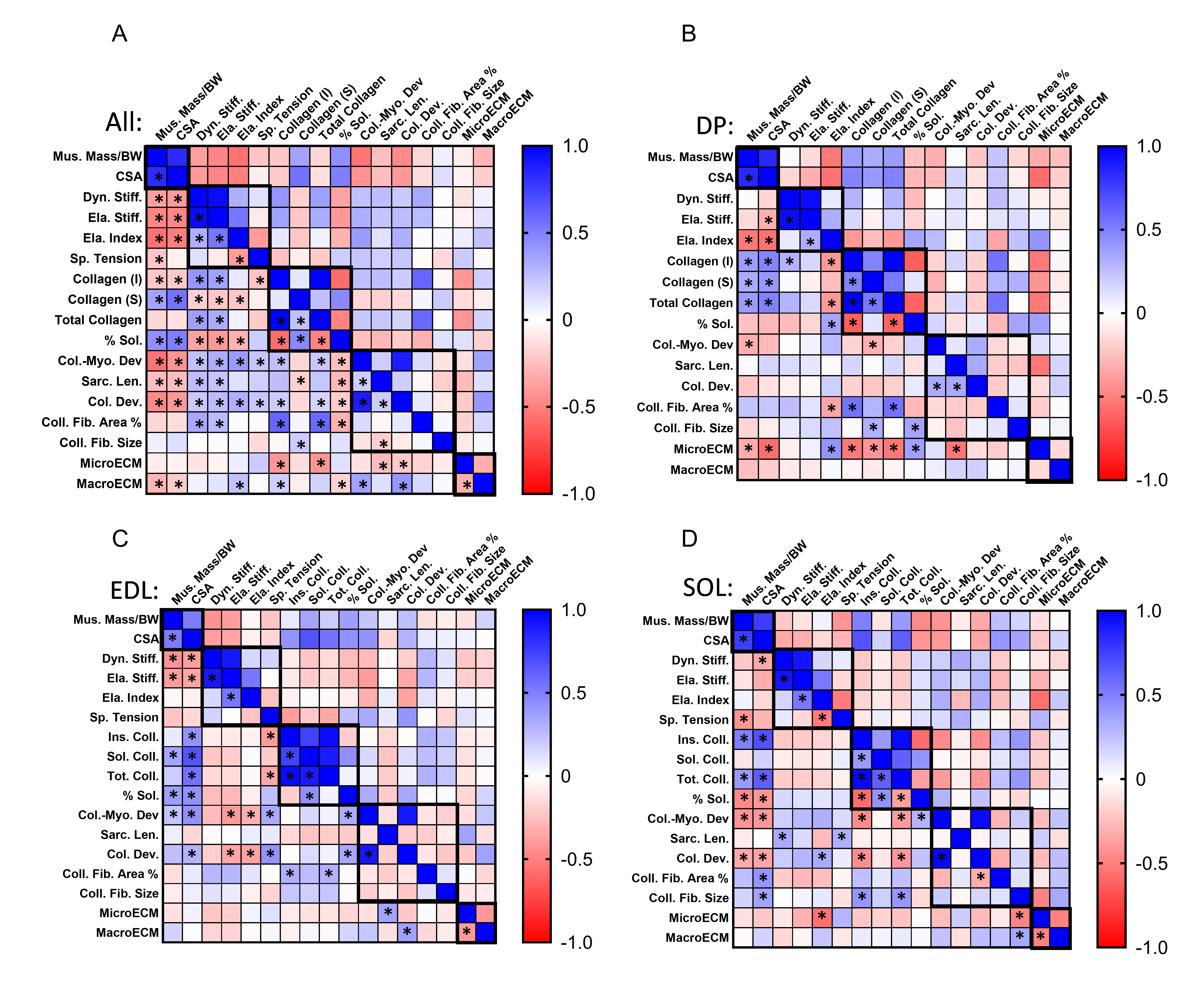

Supplement: S4 Fig — (A) Correlation matrix showing significant relationships between parameters across all muscles. (B-D) Correlation matrices of individual muscles showing significant relationships between parameters in diaphragm, EDL, and soleus muscles. *p<0.05, determined by Pearson correlation analysis. (TIF) [file pone.0271776.s004.tif]

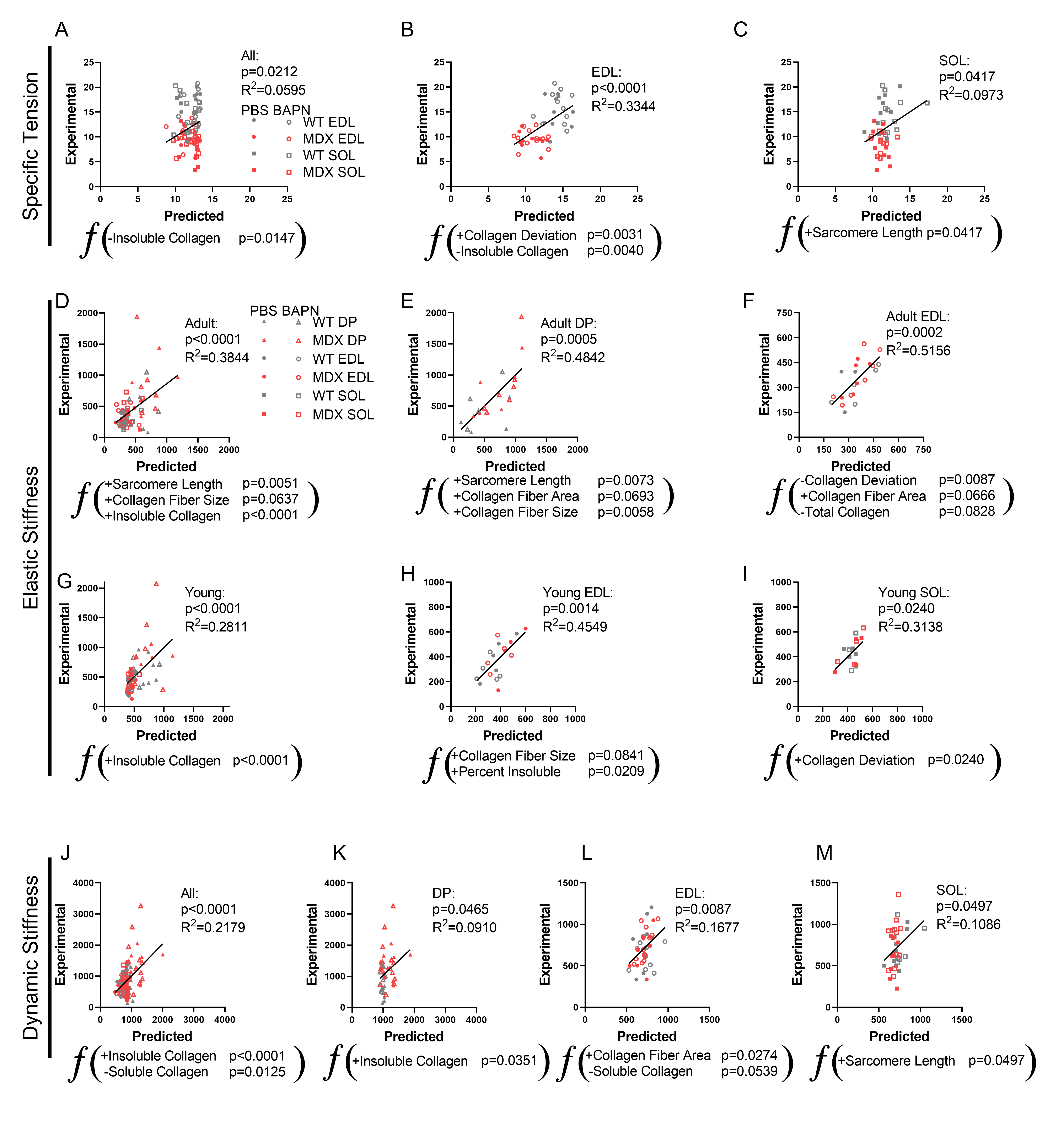

Supplement: S5 Fig — (A) The combined model produced insoluble collagen as a negative predictor of specific tension. (B) The model gave collagen deviation as a positive predictor for specific tension in EDL and insoluble collagen as a negative predictor. (C) The model gave sarcomere length as the only positive predictor for specific tension in soleus. (D-I) Multiple linear regressions of young and adult groups of muscles demonstrated significant predictors of elastic stiffness. (J-M) Multiple linear regressions of combined and individual muscles showed collagen architecture relates to dynamic stiffness. (TIF) [file pone.0271776.s005.tif]
